# A sixteen-element dual band compact array antenna for ISM/Bluetooth/Zigbee/WiMAX/WiFi-2.4/5/6 GHz applications

**DOI:** 10.1016/j.heliyon.2022.e11675

**Published:** 2022-11-24

**Authors:** Liton Chandra Paul, Md. Hossain Ali, Tithi Rani, Himel Kumar Saha, Md. Tanvir Rahman Jim

**Affiliations:** a Dept. of EECE, Pabna University of Science and Technology, Bangladesh; b Dept. of ETE, Rajshahi University of Engineering and Technology, Bangladesh

**Keywords:** Microstrip patch array antenna, ISM, Bluetooth, Zigbee, WiMAX, WiFi-2.4/5/6 GHz

## Abstract

A sixteen-element dual band microstrip array antenna with four branches has been presented in this paper. The dual band array antenna is suitable for ISM/Bluetooth/Zigbee/WiMAX/ WiFi-2.4/5/6 GHz applications. The antenna is made up of eight circular patch (r = 2 mm) elements and eight rectangular patch (1 × 8 mm) elements that are connected together to provide double band and create a suitable radiation performance with a wide bandwidth. Therefore, the array is a combination of heterogeneous elements. Initially, to justify the antenna performance that means for estimating antenna gain, directivity, E-field, H-field and system efficiency both time domain (TD) and frequency domain (FD) solvers of computer simulation technology (CST) are used. The area of the antenna is 40 × 40 × 0.79 mm^3^. Rogers RT 5880 (lossy) is used as a substrate and metal (copper) is used as radiating layers. The estimated results attest the proposed array antenna work on dual band 2.20–3.18 GHz and 4.81–7.21 GHz with centre operating frequencies of 2.54 GHz and 5.64 GHz, respectively. It maintains return loss lower than -10 dB with a better gain and directivity over the both working bands. The key objective of using the array antenna is to get the improved gain compact array antenna as well as to get dual band so that it can be used in multiple applications in daily life. The performance of the proposed array antenna is validated by three professional 3D electromagnetic simulators: high-frequency structure simulator (HFSS), FEKO (a computational electromagnetics software) and CST.

## 1 Introduction

Wireless communication is a fast-growing communication sector which has a great influence to improve the standard of living of people [1]. In the field of communication, new technologies have been added from the first to the current generation to ensure the quality of life of folk. Microstrip patch antenna has added a new dimension to the growth of wireless communication [2]. It generally consists of three layers: a substrate with a fixed dielectric constant, metallic patch and ground layer. In the upcoming days, multiband antennas will become much more significant since they have the ability to be used for multifaceted purposes. Therefore, it is already being used in several countries to serve different purposes [3]. However, highly efficient antennas having different kinds of radiation patterns are needed to achieve the dual or multiband applications [4]. Due to the pressing need of wideband, multi-frequency and multi-function antennas in wireless technologies, compact and easily integrated antennas have created a great attention in the past few years and some designs are introduced in [5, 6, 7, 8, 9, 10]. During design of a dual or multiband antenna, an antenna designer should bear in mind that the size of the antenna must be compact as much as possible and less use of metal and antenna must have intended bandwidth coverage, high radiation efficiency, good gain as well as directivity [11]. One vital challenge of deploying wireless communication systems is multipath fading which causes distortion of the base signal. Since the signal propagates through different paths as a result the traveling lengths of the signal are varied i.e. the transmitted signal arrives at the receiver over a spread of times. It creates phase distortion and inter symbol interference (ISI) during data transmission. One can overcome the multipath fading effect by using a directive antenna system or multi-input multi-output (MIMO) technology [12]. The mutual coupling effect of a MIMO antenna weakens the accomplishment of the antenna system. It not only influences the radiation efficiency but also affects the correlation. Many researchers have provided different ways to minimize mutual coupling such as defected ground structure (DGS) based ground layer connected with neutral line [13], by using parasitic elements which provide reverse coupling [14] that help to remove mutual coupling. In previously published works, various designs have been introduced like 1 × 2 microstrip patch antenna array for 5 GHz WiFi [15], dual L-shaped antenna for WLAN [16] and dual U-shaped antenna for WiMAX applications [17]. Though these antennas cover a massive room providing good multiband performance, authors designed complex structures discussed in [18, 19, 20]. A coplanar waveguide (CPW) fed dual band (BW: 0.3 GHz and 0.775 GHz) planar antenna has been discussed in [21]. In [22], the authors provide a novel dual-band U-slotted square-shaped patch antenna whose gain is 1.37 dBi and 4.37 dBi at 2.45 GHz and 5.8 GHz, respectively. CPW fed U-slotted dual band circular patch antenna is presented for 4G LTE and WiFi communications [23]. A recurrent fractal-ring patch is placed on the dielectric layer combined with another uniform fractal ring which is connected with a Y-shaped fed line to get a dual band [24]. It provides good results but the overall design process is complicated. A slotted ground antenna is introduced in [25] with a single band (2.4–2.49 GHz) and it provides return loss of -19.5 dB. A rectangular patch antenna is mentioned in [26] where overall gain and directivity is almost same (4.5 dB) which is suitable for industrial, scientific and medical (ISM) applications. A low profile triple T-topped patch antenna (34 × 24 × 0.79 mm^3^) having a gain of 2.199 dB at 3.6 GHz has been introduced in [27] for 5G and WiMAX. Another rectangular slotted sickle shaped monopole antenna is proposed in [28]. But the 2nd operating band of the antenna covers only 5.5–6 GHz and the overall dimension is slightly high. In [29], a CPW-fed novel monopole antenna shows lower gain (1.6 dB and 2 dB). An open square loop-slotted antenna at 5.5 GHz for WiFi is proposed in [30] where modified gain at the final stage is 4.25 dB at 5.5 GHz and it provides only single band. Antenna with rectangular patch is presented in [31] with a good gain and directivity which is applicable for Wi-Fi and WiMAX Applications. P.M. Mpele et al presented a FR-4 based quad band planar antenna using shorting pin, DGS and multi-branch approaches for multipurpose communication including ISM, WLAN and WiMAX [32].

In this paper a sixteen-element dual band planar array antenna with four branches is proposed for ISM, Bluetooth, Zigbee, WiMAX and WiFi-2.4/5/6 GHz applications. The rest of the article is presented as follows: the design of the sixteen-element compact array antenna is narrated in section II. The estimated results, performance evaluation and validation of the antenna model are described in section III. At last, in section IV, concluding remarks and discussion including some strong points of the array antenna are presented.

## 2 Sixteen-element compact array antenna

**Figure 1:**
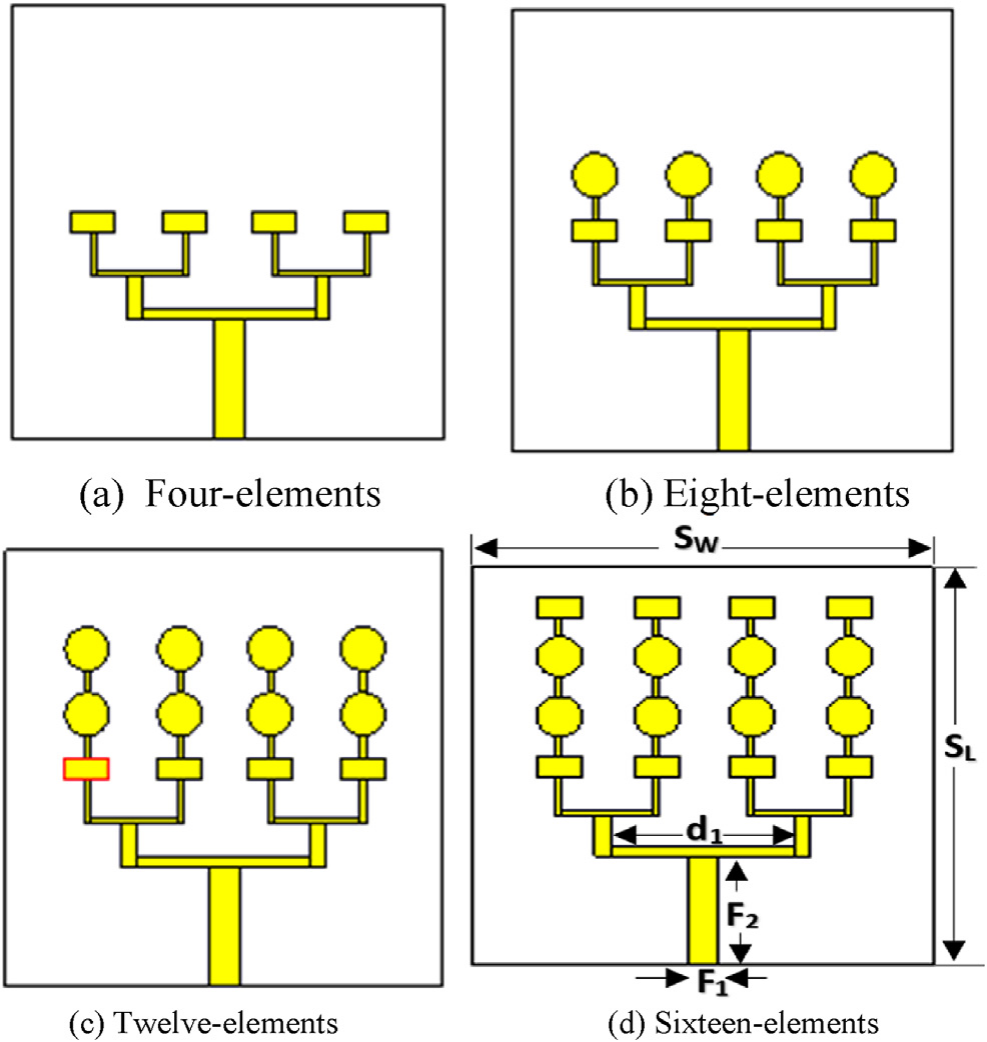
Design evolution.

The evolution steps of the designed sixteen-element microstrip array antenna is sketched in Figure 1(a-d). All the antennas are etched on a 40 × 40 mm^2^ Rogers RT 5880 (ℇ_r_ = 2.2, tanδ = 0.0009 and thickness = 0.79 mm). A standard thick (0.035 mm) radiating copper material is considered for the designed prototype. The proposed antenna consists of eight circular elements and eight rectangular elements which are connected together by using a corporate-feed network. Therefore, the array consists of two heterogeneous elements. The vertical interval between individual patch elements and the horizontal gap between the parallel branches are adjusted to avoid mutual coupling effect and grating lobes, respectively [33]. Initially, the size of the antenna has been estimated by using some fundamental Eqs. (1), (2), (3), and (4) then the greatness of the MPA is optimized to 40 × 40 × 0.79 mm^3^ by using CST microwave studio (MWS).

(1) $$Patch\;width,W_{P\;}=\frac{c}{2f_{r}}\sqrt{\frac{2}{\varepsilon_{r}+1}}$$

where, *c* = Velocity of light, *f*_*r*_ = Resonance frequency and *ε*_*r*_ = Dielectric constant

(2) $$Length,L=0.412h\frac{\left(\varepsilon_{reff}+0.3\right)\left(\frac{W_{p}}{h}+0.264\right)}{\left(\varepsilon_{reff}-0.258\right)\left(\frac{W_{p}}{h}+0.8\right)}$$

(3) $$Effective\;length,L_{eff}=\frac{c}{2f_{r}\sqrt{\varepsilon_{reff}}}$$

And

(4) $$Patch\;length,L_{p}=L_{eff}-2\Delta L$$

To analyse the function of circular elements and rectangular elements as well as to reduce the design complexity of the array antenna, we kept the radius of the circular patches and area of the rectangular patches fixed. The radius of the circular element is 2 mm and the length and width of the rectangular element are 4 mm and 2 mm, respectively, that are connected together by using a metallic strip line.

**Figure 2:**
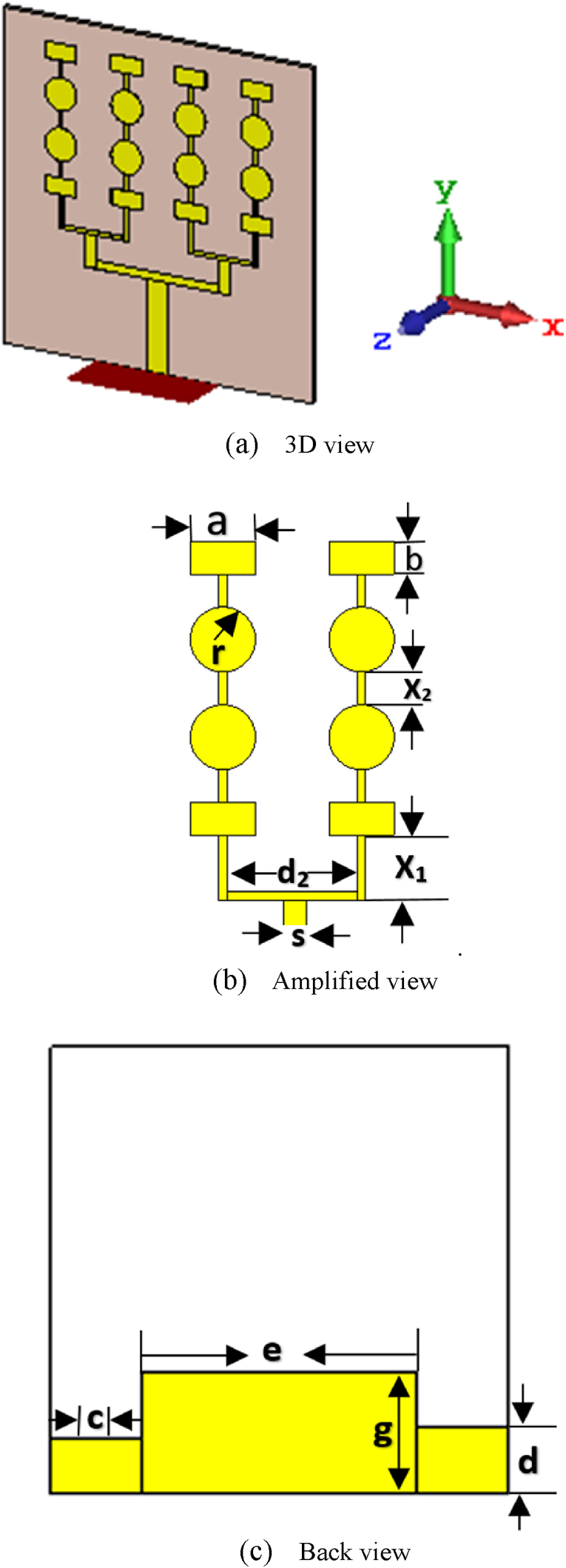
Proposed sixteen-element array antenna.

The radius of the circular elements and size of the rectangular patch have been selected after doing a number of experiments in CST-MWS 3D simulation software so that we can acquire the desired frequency bands covering ISM/Bluetooth/Zigbee/WiMAX/WiFi-2.4/5/6 GHz applications. The inclusion of a rectangular element at the top of each radiating branch of the antenna provides large dual band coverage as well as good performance for the intended multiple applications. The feed line width and height are 2.4 mm and 11 mm, respectively. The feed line width is selected in such a way so that input impedance is matched properly. The complete structure of the proposed sixteen-element array antenna including necessary dimensional labelling has been presented in Figure 2 (a-c). The estimated dimension matrices of the proposed sixteen-element array antenna are depicted in Table 1.

**Table 1:** List of dimension matrices.

Name and denotation	Value (mm)
Distance between two strip line (d_1_)	16
Feed's width (F_1_)	2.4
Feed's length (F_2_)	11
Length of rectangular element (a)	4
Width of rectangular element (b)	2
Radius of circular element (r)	2
Length of feed line of first element of each branch (x_1_)	4
Length of strip line between two successive patch elements (x_2_)	2
Distance between two adjacent branches (d_2_)	8
Width of strip (s)	0.5
Width of small block of ground plane (c)	8
Length of small block of ground plane (d)	6
Width of large block of ground plane (e)	24
Length of large block of ground plane (g)	11

## 3 Performance evaluation

**Figure 3:**
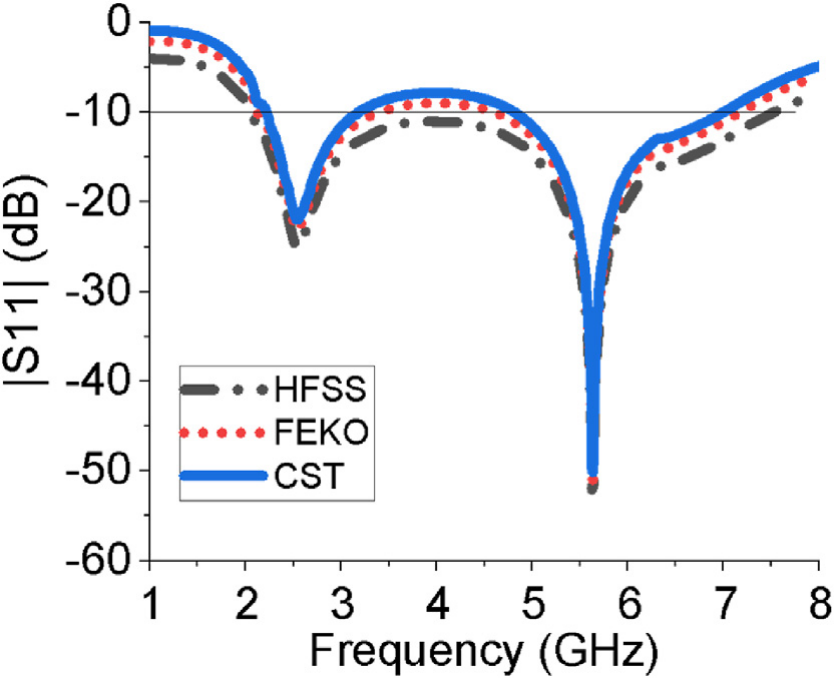
|*S*_*11*_| curve of the sixteen-element antenna estimated by HFSS, FEKO and CST.

**Figure 4:**
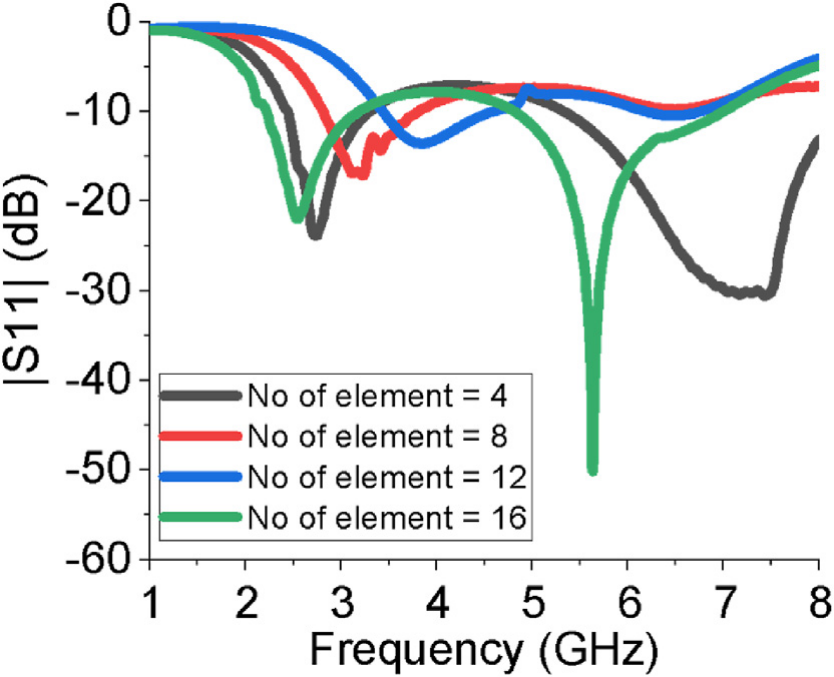
|*S*_*11*_| curve and number of elements of the antenna.

**Figure 5:**
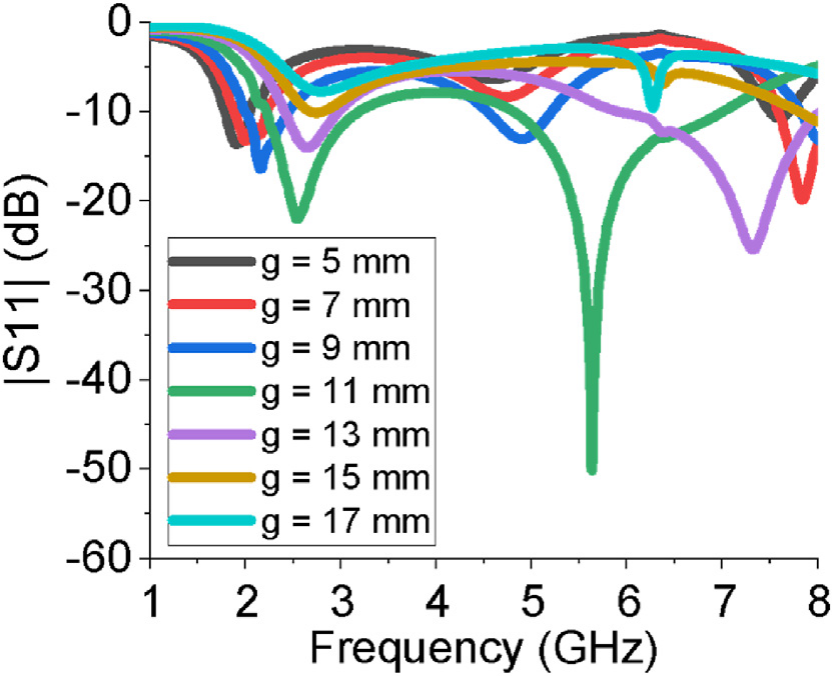
|*S*_*11*_| curve and length of large block of ground plane (g) of the sixteen-element antenna.

The performance of the sixteen-element array antenna is optimized by using CST-MWS suite. The CST helps us to estimate the antenna parameters such as |*S*_*1*1_| curve, operating frequency range, gain, directivity, VSWR, E-field, H-field, current distribution, input impedance, efficiency etc. Those parameters interpret how much the proposed sixteen-element array antenna is suitable for aforementioned real life applications. From Figure 3, the sixteen-element array provides good impedance matching over two operating bands, 2.2–3.18 GHz and 4.81–7.21 GHz, with reflection coefficient of less than -10 dB. In the case of the first band, -10 dB bandwidth is 0.98 GHz and return loss at the centre frequency of 2.54 is -24 dB. In the case of the second band, -10 dB bandwidth is 2.4 GHz and return loss is -52 dB at 5.64 GHz. In addition, it provides large bandwidth covering desired ISM/Bluetooth/Zigbee/WiMAX/WiFi-2.4/5/6 GHz services. The scattering parameter (*S*_*11*_) estimation of the designed sixteen-element antenna has also been verified by high-frequency structure simulator (HFSS) and FEKO (a computational electromagnetics software). All the results derived from three different simulators show very good agreement among them. The |*S*_*11*_| response of the array has been also analysed by changing the number of elements in the array antenna as presented in Figure 4. The sixteen-element array responds properly to tune dual operating bands at all the intended ISM/Bluetooth/Zigbee/WiMAX/WiFi-2.4/5/6 GHz communication. The impact of the variation of the length of the large block of ground plane (g) is studied in Figure 5 which vividly shows the array antenna provides the best reflection coefficient for g = 11 mm.

**Figure 6:**
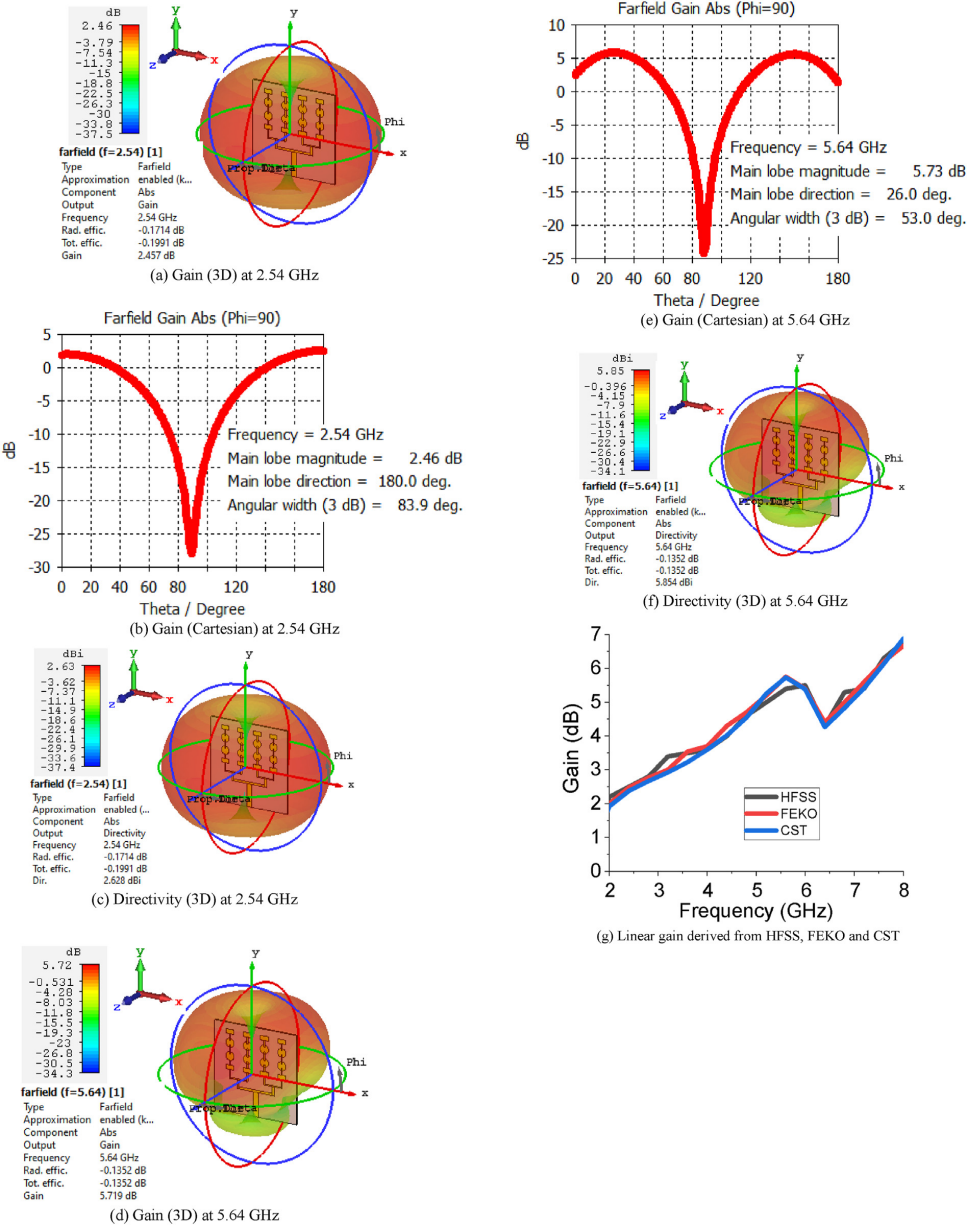
Gain and directivity of the sixteen element array antenna.

**Figure 7:**
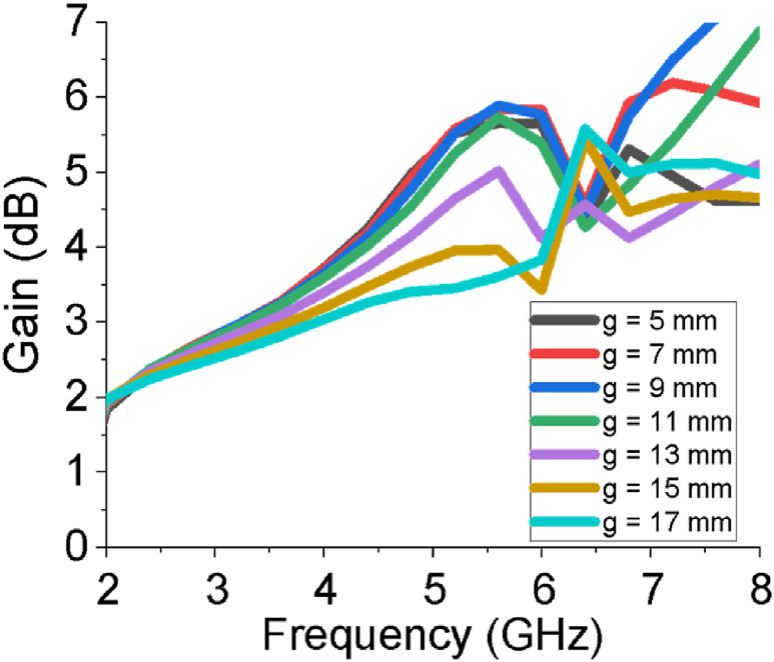
Impact of length of large block of ground plane (g) on gain of the antenna.

**Figure 8:**
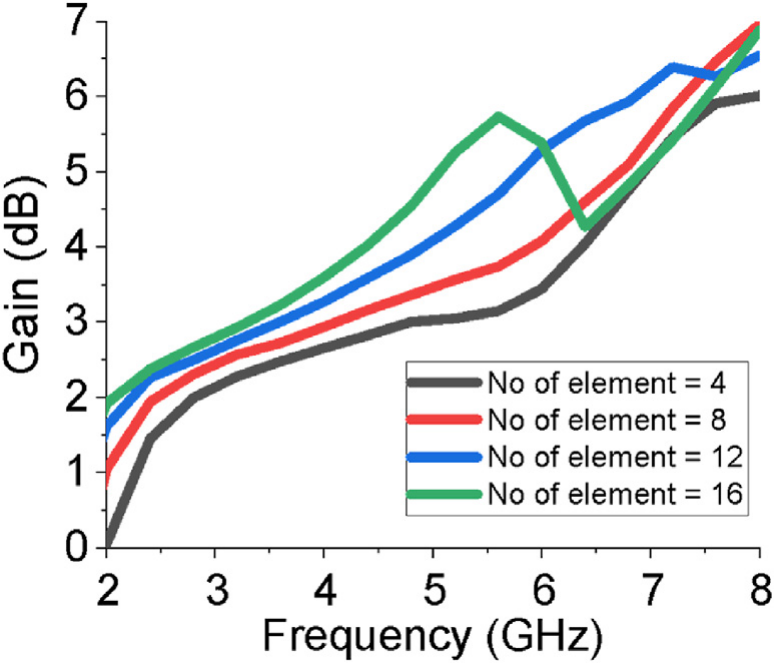
Impact of number of elements of the array on gain of the antenna.

The gain and directivity curves of the sixteen-element array antenna in different forms (3D, Cartesian and linear) are presented in Figure 6(a-g). The estimated gain of the heterogenious array antenna is 2.45 dB at 2.54 GHz and 5.71 dB at 5.64 GHz, and directivity is 2.62 dBi at 2.54 GHz and 5.85 dBi at 5.64 GHz. The linear gain of the array is varied from 2.1 dB to 5.71 dB as in Figure 6(g). The estimated gain of the array antenna is also buttressed by HFSS and FEKO. The role of the large block span of the ground plane (g) and the number of elements of the array on gain are studied and presented in Figures 7 and 8, respectively. From the investigation, the overall gain is good in the both operating bands for g = 11 mm and sixteen elements of the array antenna. The gain of the designed sixteen-element array antenna increases with increasing the number of elements which supports the basic principle of an array antenna.

**Figure 9:**
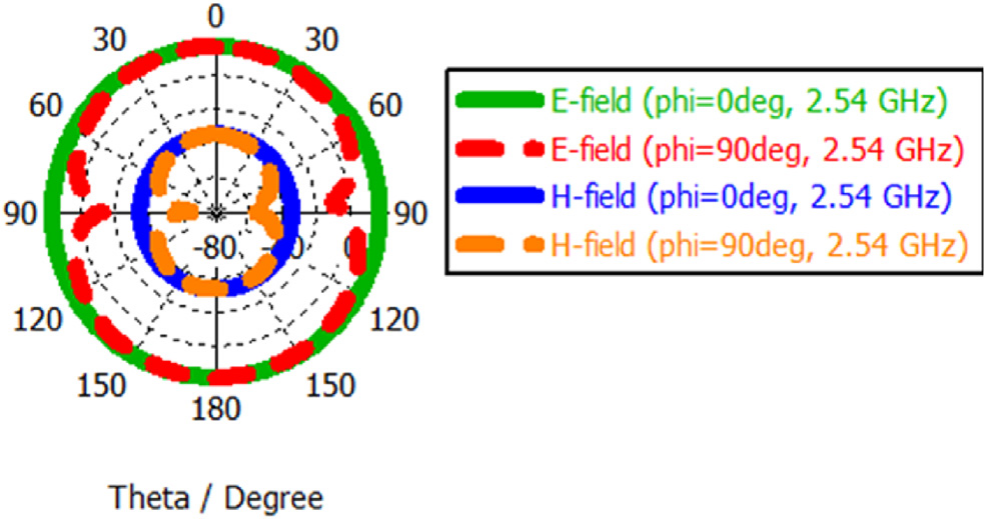
E-field and H-field curve at 2.54 GHz.

**Figure 10:**
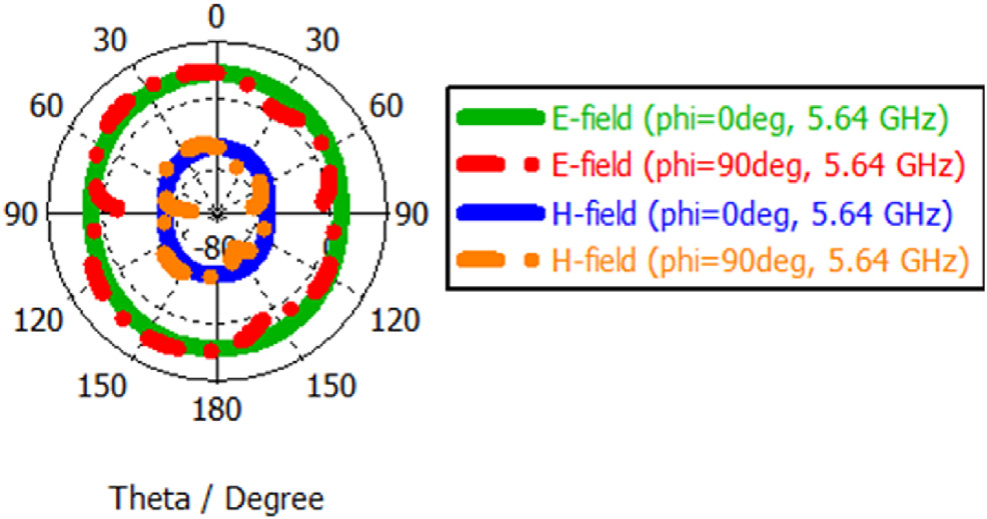
E-field and H-field curve at 5.64 GHz.

The polar form radiation patterns at 2.54 and 5.64 GHzare shown in Figures 9 and 10, respectively. The compact sixteen-element array exhibits omnidirectional radiation in the E-plane at phi = 0° and bidirectional pattern in the E-plane at two resonant working frequencies which is needed for afore-discussed handheld applications. The radiation pattern of an antenna explicates the 3dB angular beamwidth, main lobe magnitude, the direction of the main lobe and the side lobe level. In the E-field, the main lobe magnitude at φ = 0° is 17.2 dBV/m and the 3 dB angular beam width at φ = 90° is 85.9° at 2.54 GHz. In the case of H-field, the main lobe magnitude at φ = 0° is -34.3 dBA/m and the 3 dB angular beam width at φ = 90° is 85.9° at 2.54 GHz.

At the 2^nd^ operating band, the prime lobe magnitude at φ = 0° is 17.2 dBV/m and the 3 dB angular beam width at φ = 90° is 83.6° at 5.64 GHz. In the case of H-field, the main lobe magnitude at φ = 0° is -34.3 dBA/m and the 3 dB angular beam width at φ = 90° is 53.0° at 5.64 GHz.

**Figure 11:**
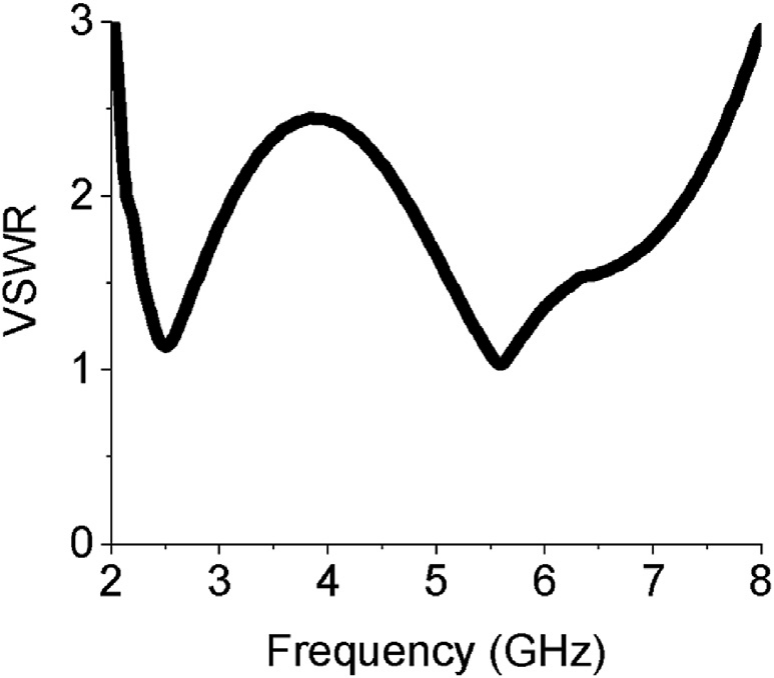
VSWR of the array antenna.

**Figure 12:**
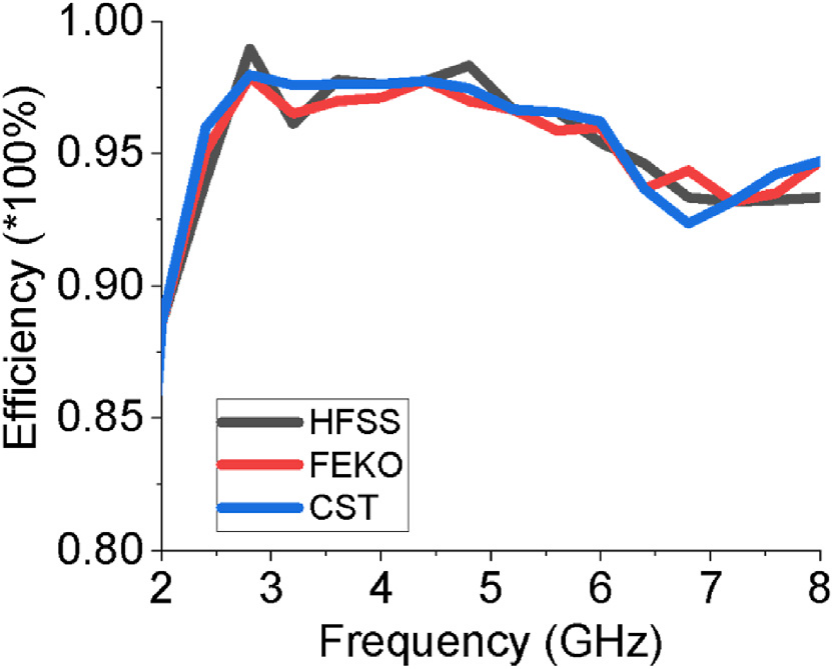
Radiation efficiency of the antenna.

**Figure 13:**
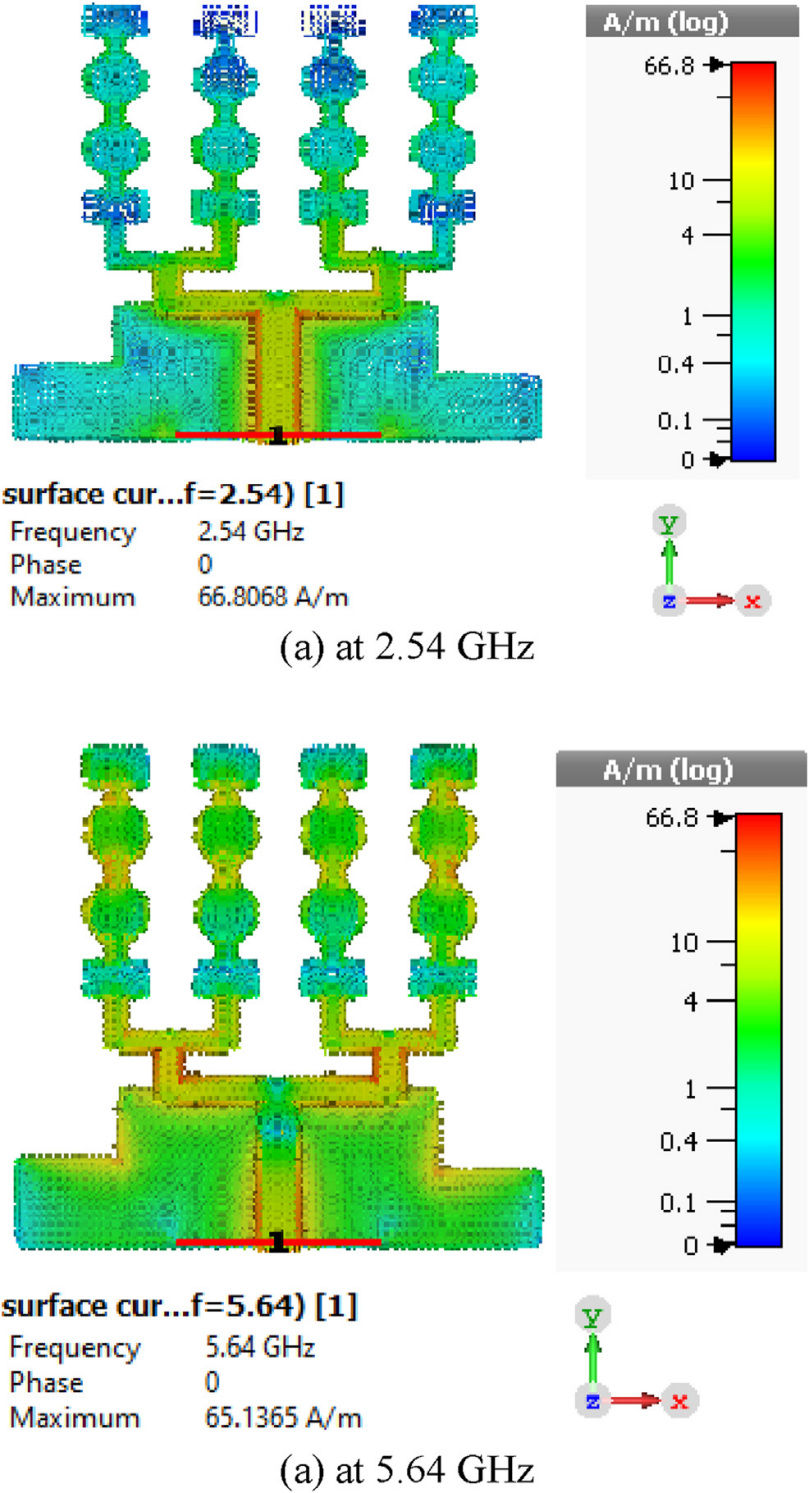
Surface current distribution.

The VSWR of the array antenna is given in Figure 11 which alludes that the designed sixteen-element array antenna has VSWR of 1.17 at 2.54 GHz and 1.006 at 5.64 GHz that delivers favorable impedance matching. For VSWR ≤2, the achieved first working band lies between 2.20 to 3.18 GHz, while the second one is within 4.81–7.21 GHz. The average efficiency of the sixteen-element array is above 95% which is another strong feature for the proposed sixteen-element design. The efficiency estimated by CST of the antenna is buttressed by HFSS and FEKO as shown in Figure 12. The surface current at 2.54 GHz is 66.8086 A/m and at 5.64 GHz is 65.1365 A/m as presented in Figure 13. In Figure 13 (a) current density is slightly higher at the lower part of the feed line which is responsible for the first lower frequency band (2.20–3.18 GHz). The current density gets higher as shown in Figure 13(b) at the edges of upper parts of the patch elements and sub-feeder for higher operating band (4.81–7.21 GHz).

**Figure 14:**
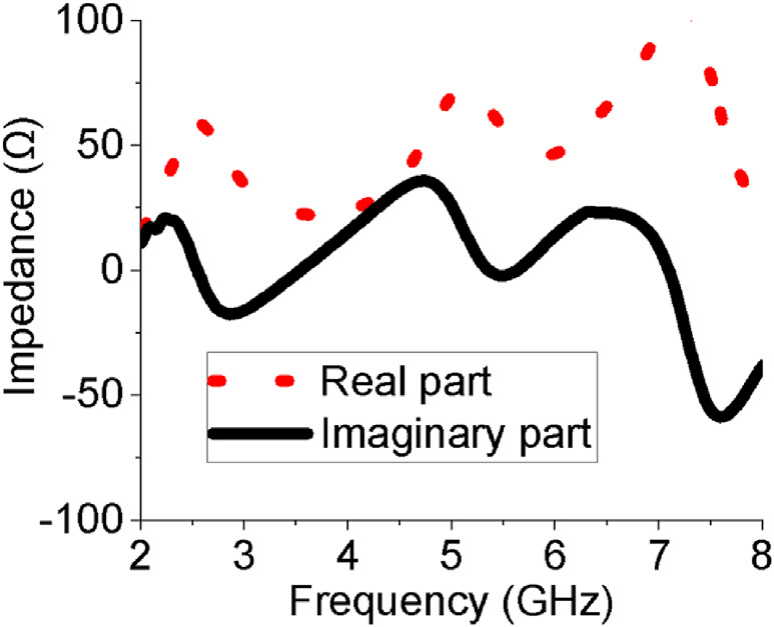
Z – Parameters of the sixteen-element array antenna.

Figure 14 represents the Z-parameters of the array antenna where real part and imaginary part at 2.54 and 5.64 GHz are near about 50 Ohm and 0 Ohm, respectively; that means the array antenna provides perfect impedance matching. The imaginary part of the sixteen-element array indicates that the energy stored in the near field of the antenna is a non-radiated power which is reduced by good impedance matching. Table 2 provides a summary of the radiation characteristics of the designed sixteen-element array antenna. Some recently published and relevant works have been studied and presented side by side along with our proposed array antenna in Table 3. The designed model of the sixteen-element array antenna exhibits larger bandwidth than all other works keeping compactness, excellent reflection coefficient profile as well as gain. Since the array antenna is highly efficient, it would be more suitable for a wide range of handheld applications like ISM/Bluetooth/Zigbee/WiMAX/WiFi-2.4/5/6 GHz.

**Table 2:** Results of dual band patch array antenna.

Description	Value	
	1^st^ band	2^nd^ band
Lower cut off	2.20 GHz	4.81 GHz
Upper cut off	3.18 GHz	7.21 GHz
Bandwidth (BW)	0.98 GHz	2.40 GHz
Resonant point	2.54 GHz	5.64 GHz
Reflection coefficient	-24 dB	-52 dB
VSWR	1.17	1.006
Gain (Max.)	2.45 dB	5.71dB
Directivity (Max.)	2.62 dBi	5.85 dBi
Maximum radiation efficiency	97%	96%
Real part of impedance	58.88 Ω	50.3 Ω
Imaginary part of impedance	0.99 Ω	0.4 Ω

**Table 3:** Comparison table.

Parameter	Reference No.										This work
	[28]	[30]	[31]	[34]	[35]	[36]	[37]	[38]	[39]	[40]	
Size (L×W×h) mm^3^	60 × 40×0.8	19 × 20×1.6	40 × 40×1.5	28 × 43×0.2	22 × 24×1.59	40 × 44×∗	38 × 50×3.048	22 × 28×1.5	46 × 46×3.175	150 × 80×0.8	40 × 40×0.79
Substrate material	FR-4	FR-4	FR-4	Polyimide	FR-4	Rogers RO4003	Rogers RO4350B	FR-4	Rogers RT	FR-4	RogersRT5880
Operating Frequency Range (GHz)	1.8–3.1, 5.5–6	5.47–5.87	2.314–2.492, 5.586–6.06	2.3–3.8, 4.5–5.1, 6.3–6.8	2.4–2.74, 3.25–3.64	2.30–2.98, 5.13–7.75	5.64–5.8	5.25–5.75	2.44–2.54, 3.19–3.55	∼1.27–1.5, ∼2.2–2.5, ∼3.5–4.2	2.2–3.18, 4.81–7.21
Centre frequency (GHz)	2.7, 5.7	5.5	2.4, 5.8	∼3, ∼4.8, ∼6.4	2.48, 3.49	2.5, 5.6	5.7	5.5	2.5, 3.2, 3.45	∼1.3, ∼2.4, ∼3.8	2.54, 5.64
Return loss (dB) at centre frequency	-42, -45	≈ -37	-32.7, -25.9	∼ -20, ∼ -25, ∼ -20	-13.2, -32.67	-41.5, -32.91	∼ -25	∼ -20	∼ -22	∼ -18, ∼ -18, ∼ -35	-24, -52
BW (GHz)	1.3, 0.5	0.4	0.178, 0.474	1.5, 0.6, 0.5	0.34, 0.39	0.68, 2.62	0.16	0.5	0.1, 0.36	0.23, 0.3, 0.7	0.98, 2.4
Gain (dB)	2.6, 4.6	4.25	3.4, 4.5	4	2.4, 3.5	NA	5.8	NA	∼4	5	2.62, 5.85

● NA = Not available.

## 4 Conclusion

A dual band sixteen-element array antenna has been exhibited in this paper whose design is compatible for multiple wireless communication applications like ISM/Bluetooth/Zigbee/WiMAX/WiFi-2.4/5/6 GHz. The main benefit of the antenna is it's suitable for ISM (2–2.4 GHz), Zigbee (2.407–2.484 GHz), Bluetooth (2.407–2.484 GHz), WiMAX rel 1 (2.3–2.4 GHz), WiMAX rel 1.5 (2.5–2.69 GHz), WiFi 2.4 GHz (2.407–2.48 GHz), WiFi 5 GHz (5.15–5.85 GHz) and WiFi 6 GHz (5.925–7.125 GHz) services. Some other advantages of the proposed array antenna are structural simplicity, large bandwidth, excellent radiation coefficient profile, higher efficiency as well as compactness. The various radiation properties at different centre frequencies make the submitted antenna versatile and can be used for different communication systems. The impact of the number of elements of the array and span of the partial ground plane have also been studied. The performance of the heterogeneous sixteen-element array has been estimated and buttressed by the three professionally available 3D electromagnetic software: HFSS, FEKO and CST. After investigating and evaluating the properties of the sixteen-element array antenna, it could be a new candidate for the aforementioned wireless services.

## Declarations

### Author contribution statement

Liton Chandra Paul; Md. Hossain Ali: Conceived and designed the experiments; Performed the experiments; Analyzed and interpreted the data; Contributed reagents, materials, analysis tools or data; Wrote the paper.

Tithi Rani; Himel Kumar Saha; Md. Tanvir Rahman Jim: Performed the experiments; Analyzed and interpreted the data; Contributed reagents, materials, analysis tools or data; Wrote the paper.

### Funding statement

This research did not receive any specific grant from funding agencies in the public, commercial, or not-for-profit sectors.

### Data availability statement

Data included in article/supp. material/referenced in article.

### Declaration of interest's statement

The authors declare no conflict of interest.

### Additional information

No additional information is available for this paper.
